# CATS: a bioinformatic tool for automated Cas9 nucleases activity comparison in clinically relevant contexts

**DOI:** 10.3389/fgeed.2025.1571023

**Published:** 2025-07-24

**Authors:** Ettore Rocchi, Federico Magnani, Gastone Castellani, Antonio Carusillo, Martina Tarozzi

**Affiliations:** ^1^Department of Medical and Surgical Sciences, University of Bologna, Bologna, Italy; ^2^ IRCCS University Hospital of Bologna, Bologna, Italy; ^3^ Alia Therapeutics, Trento, Italy

**Keywords:** CRISPR/Cas9, PAM, bioinformatics, genome editing, bioinformatic software, PAM comparison, allele-specific

## Abstract

**Introduction:**

With the growing number of Cas9 nucleases available to genetic engineers, selecting the most suitable one for a given application can be challenging. A major complication arises from the differing protospacer adjacent motif (PAM) sequence requirements of each Cas9 variant, which makes direct comparisons difficult. To ensure a fair comparison, it is essential to identify common target sites that are not biased by the natural genetic landscape of the chosen target.

**Methods:**

To address this challenge, we developed CATS (Comparing Cas9 Activities by Target Superimposition), a novel bioinformatic tool. CATS automates the detection of overlapping PAM sequences across different Cas9 nucleases and identifies allele-specific targets, particularly those arising from pathogenic mutations. One of the key parameters in CATS is the proximity of PAM sites, which helps minimize sequence composition bias. The tool integrates data from continuously updated sources and includes ClinVar information to facilitate the targeting of disease-causing mutations.

**Results:**

CATS significantly reduces the time and effort required for CRISPR/Cas9 experimental design. It streamlines the comparison of Cas9 nucleases with different PAM requirements, enabling researchers to select the most appropriate nuclease for their specific target. The tool’s automation, speed, and user-friendly interface make it accessible to researchers regardless of their computational expertise.

**Discussion:**

By enabling the identification of overlapping PAMs and allele-specific targets, CATS supports the implementation of Cas9-based applications in both research and clinical settings. Its ability to incorporate genetic variants makes it particularly useful for designing therapeutic approaches that selectively target mutated alleles while sparing healthy ones. Ultimately, CATS contributes to the development of more effective and precise genetic therapies.

## 1 Introduction

The CRISPR-Cas system offers the unparalleled ability to target any location within the genome by simply changing a 20-23 nucleotides (nt) sequence, known as the spacer, of the single guide RNA (sgRNA). This provides a significant advantage over other genome editing tools, such as ZFNs and TALENs, which require more lengthy and complex re-engineering of the DNA binding domain (Gupta and Musunuru, 2014). To function, the Cas9:sgRNA complex requires the presence of a Protospacer Adjacent Motif (PAM) at the genomic location specified by the spacer (Yuan, 2025). The PAM is a short DNA sequence of 3-8 nucleotides (nt) essential for Cas9 nucleases’ binding and activity. This has driven the discovery of novel Cas9 enzymes with different PAM requirements compared to the gold-standard NGG-PAM SpCas9 (e.g., SaCas9 (Ran et al., 2015) like NmCas9 (Lee et al., 2016), CjCas9 (Kim et al., 2017)) or near-PAMless Cas9 enzymes (SpRY) (Liang et al., 2022). This, along with the need to develop smaller nucleases that can fit into viral vectors, has led to a large portfolio of nucleases. Faced with this large variety, it can be challenging to choose the right one. When size is not a discriminating factor, cleavage activity becomes the main bottleneck. The bioinformatic tools currently available (e.g., Cas-designer (Park et al., 2015), CHOPCHOP (Labun et al., 2019) and CRISPOR (Concordet and Haeussler, 2018)) have been designed with the intent to provide the user with a list of gRNAs for a specific nuclease targeting a gene of interest (GOI). Here, the main output is a list of gRNAs with their predicted efficiency, provided as indel frequency. Such tools therefore are used when the scientist wants, for example, to knock-out a gene and wishes to short-list the gRNAs with the highest predicted efficiency among all the possible ones.

However, when developing new genome editing-based approaches, a researcher may need to compare two Cas9 nucleases in the same genomic context, rather than finding the most efficient gRNA for a specific nuclease. This is particularly important when a new Cas9 nuclease is described and needs to be compared against the gold-standard. Nucleases’ activity is not simply an inherent quality, but it strongly depends on the guide-RNA sequence (Moreb and Lynch, 2021) and the genomic target (Corsi et al., 2022). Therefore, different Cas nucleases cannot simply be directly compared to each other by targeting the same gene. Ideally, a more stringent approach would require two nucleases targeting different genes (5 to 10) at different positions (e.g., 2 to 3 sgRNAs) (Pedrazzoli et al., 2024). Normally, this would involve manual curation, which is time-consuming and limits scalability and applicability in larger-scale genomic studies (e.g., across panels of different targets). Therefore, a robust method for detecting and analyzing these overlaps is essential to fully tap into the potential offered by the wide variety of programmable Cas9 nucleases currently available.

Having this angle in mind, we developed Comparing Cas9 Activities by Target Superimposition (CATS), a bioinformatic tool designed to automatically detect genomic regions where two PAM sequences overlap and provide comprehensive annotation of pathogenic mutations within the 25 nucleotides–up- and down-stream–proximal to the overlapping PAM sequences. This tool addresses the need for more efficient and automated comparison of Cas9 nucleases by overlapping PAM sequences scanning. By automating the detection of overlapping PAM sequences, our tool significantly reduces the time and effort required for analysis, making it accessible to a broader range of researchers.

As mentioned above, CRISPR-mediated DNA cleavage requires the PAM sequence to be next to the intended target. If the PAM is absent or mutated, the CRISPR-complex cannot cleave the DNA. This has been exploited to address autosomal-dominant conditions. In this context, mutations in one allele may cause a dominant-negative phenotype which is enough to impair the healthy-allele function and trigger the condition. Because of this, researchers have envisioned that disrupting the mutated allele may ameliorate or cure the disease. For the approach to work, the pathogenetic SNP would generate a *de novo* PAM enabling the discrimination of the two alleles (Caruso et al., 2022; Wu et al., 2020). For example, in the context of the Hyper-IgE-Syndrome (König et al., 2022), Huntington’s disease (Shin et al., 2016), Retinitis Pigmentosa (Diakatou et al., 2021) and Epidermolysis Bullosa (Cattaneo et al., 2024). Prompted by this compelling application, we implemented in CATS the possibility to cross-talk with ClinVar (Landrum et al., 2018), so that it would provide–upon request–the pathogenic mutations within the genetic regions investigated by the user. Particularly, CATS would highlight the mutations which can either result in a *de novo* PAM or would occur in the first 10 nt (default value, different numbers of nt can be set by the user) before the PAM, the so called “seed-sequence” (Baranova et al., 2024). Both alterations can be leveraged to discriminate between the two alleles. This feature will greatly facilitate the researchers wishing to implement allele-specific approaches for the treatment on autosomal-dominant conditions characterized by a detrimental gain-of-function (e.g., dominant negative) associated with the mutated allele. CATS offers a significant improvement in genome editing research by efficiently automating the detection of shared PAM sequences for different Cas9 nucleases. This capability, combined with its integration of ClinVar annotations, allows for the targeted comparison of nucleases within clinically significant genomic regions, contributing to accelerating the development of precise therapeutic interventions.

## 2 Results

### 2.1 Tool description

CATS enables the analysis of genomic sequences to identify regions where two PAM sequences of interest appear in proximity or overlap. It also supports the integration of genetic variants, thereby allowing the investigation of clinically relevant scenarios. In this way, the tool allows researchers to determine if - and which - variants would enable allele-specific targeting, either because they result in the *de novo* generation of a PAM or because of a mutation in the seed-sequence before the specified PAM. Known pathogenic mutations are retrieved from the ClinVar database (Landrum et al., 2018). CATS comes with built-in references for human and mouse genomes, based on GENCODE transcript sequences covering both protein-coding and full transcriptomes (human: GENCODE 47, mouse: GENCODE M36, FASTA “Transcript sequences”) (Frankish et al., 2023). The analysis can be limited to a selected set of genes, which is particularly recommended when working on mutated versions of the genome. Users working with non-standard genomes or focusing on non-coding regions of the human or murine genomes can use custom sources. If a GTF annotation file is provided alongside the FASTA file, the analysis can be further refined by entering gene names. However, the ‘pathogenic’ option, which automatically screens mutated versions of the selected genome, is available only for the human genome, as it relies on ClinVar annotations. Curated variant annotations regarding non-coding portions of the genome should be provided by the user.

By default, CATS performs a transcript-agnostic search for PAM motifs across the selected FASTA sequence, based on user-defined parameters such as PAM sequence, window size, and gene list. Transcript-specific information is introduced only when the ‘pathogenic’ option is selected. In this case, CATS retrieves pathogenic variant data from ClinVar and restricts the analysis to the principal transcript defined by ClinVar for each gene, ensuring clinical relevance and consistency. CATS is optimized for coding regions due to their more established functional interpretation. Users can upload their own curated datasets to analyze specific non-coding transcripts or alternative splicing isoforms.

**FIGURE 1:**
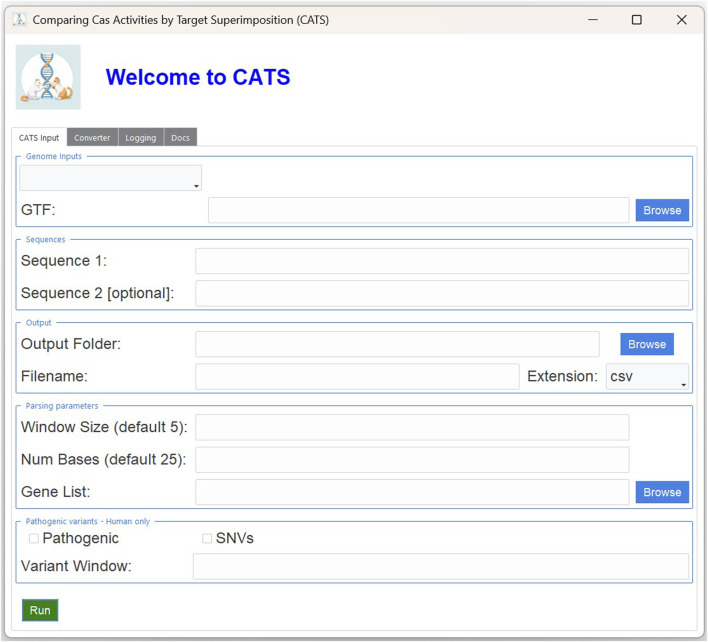
Graphical interface of CATS. In the “*Genome Inputs*” panel, the user can select from the dropdown menu one of the reference genomes retrieved from GENCODE (human full transcriptome, human protein coding, mouse full transcriptome or mouse protein coding) or provide a custom FASTA. In the “*Sequences*” panel, the user can type at least one PAM sequence to search for in the selected FASTA. In the “*Parameters*” panels, the user must select the output file name and folder. Additionally, they can change the default values of the distance between the two PAM sequences and the length of the upstream sequence to show, in the nearing of the PAM sequences of choice. Additionally, when ran with a GTF file, the user can also restrict the analysis to a list of genes of interest. The analysis of the mutant genome, which is possible only with the human reference genome, is activated by the “*Pathogenic*” option. The user can select whether to restrict the analysis to SNV mutations with the “*SNV*” option. Finally, the width of the window around the PAM sequence in which to look for mutations is specified by the “*variant window*” parameter.

The key drivers of the analysis are the nucleotide sequences of the PAM of interest, which are specified through the standard IUPAC notation reported in Table 1. CATS is not limited to a predefined set of CRISPR/Cas9 systems. Instead, it is designed to be flexible and can accept any PAM sequence as input. However, it is important to note that in its current implementation, CATS is optimized for Cas9-like systems—specifically, those in which the PAM is located immediately downstream (3′) of the spacer and where cleavage occurs approximately 3 nucleotides upstream of the PAM. Either one or two PAM sequences can be indicated, along with the amount of context to be reported in the results, i.e., the number of nucleotides before and after each sequence occurrence. If multiple sequences are given, the tool will look for their co-occurrences, which are defined by the presence of both sequences at a distance less than a given number of base pairs. The width of this window can be specified by the user. For details on the setup for performing these analyses, see Materials and Methods. Figure 1 displays the graphical interface of CATS and the options previously described.

**TABLE 1:** IUPAC notation for identifying classes of nucleotide sequences.

Symbol	W	N or *	R	Y	S	K	M	B	D	H	V	. or -
Nucleotide	AT	ACGT	AG	CT	CG	GT	AC	CGT	AGT	ACT	ACG	. or -

### 2.2 Output of the analysis

The type of information gathered by CATS depends on the usage scenario. The gene in which the occurrence has been found is always reported, along with the structural annotations of the region, the strand being used (the reference strand to employ is indicated in the annotations) and the nucleotide sequence. For each PAM, the genomic location of its occurrence is indicated. Additionally, if the search is performed activating the ‘pathogenic’ option, then the variant position, the variant name (as indicated by ClinVar) and the distance of the mutation from the PAM sequence are given, which allows to further investigate the variant. If available, its associated condition is retrieved. Table 2 reports the information gathered by CATS for the various set-up possibilities. If a custom genetic source is provided without the corresponding annotations, CATS outputs the list of the PAMs’ co-occurrences, along with the transcript’s identifier, the surrounding nucleotide bases, the PAM sequences identified and their location within the transcript. All the retrieved information is available either in tabular and in BED format, which can be inspected visually through tools such as IGV (Robinson et al., 2011).

**TABLE 2:** The information gathered by CATS, depending on the usage scenario.

Usage	2 PAMs, WT genome	1 PAM, variant genome
Transcript ID	✓	✓
Gene name	✓	✓
Regions	✓	✓
Strand	✓	✓
Sequence	✓	✓
Matched sequence	✓	✓
Matched seq. index	✓	✓
Second sequence	✓	✖
Second seq. index	✓	✖
Variant position	✖	✓
Variant distance	✖	✓
Variant name	✖	✓
Condition	✖	✓

Information about the genomic location is reported by indicating the gene, the transcript and the coordinates with respect to the transcript. The biotype of the genomic region in which the occurrence has been found is given, along with the sequence matching the IUPAC, code used for specifying the search and the nucleotidic context, comprising a custom number of base-pairs before and after the match. If the search is performed on the mutated genome, information on the variant is reported. The variant name allows further inspection in the ClinVar database (see also Table 3).

**TABLE 3:** Example of one match, from the analysis of CATS for the mutated genome, comprising the list of all the mutations happening near to a PAM, or creating a *de novo* PAM.

Transcript ID	ENST00000252242.9|ENSG00000186081.12
Gene Name	KRT5
Region	UTR5:1-98|CDS:99-1871|UTR3:1872-2238||variant_single-nucleotide-variant_NM_000424.4(KRT5):c.1396G>C
Strand	-
Sequence	CCG​GAG​CGC​GGG​CAG​TCG​TAG​CT
Matched seq	CGG
Matched seq index	chr12:52516676
Variant position	chr12:52516680-52516680
Variant distance	2
Variant name	NM_000424.4(KRT5):c.1396G>C (p.Glu466Gln)
Condition	Epidermolysis bullosa simplex

Each occurrence is annotated by the following information: the transcript to which the search result belongs, the gene to which the transcript belongs, the genomic structure annotations, the strand being used as standard, the nucleotide context in which the search result appears, the sequence matching the indicated IUPAC, expression, its location in the genome, the variant name as given by ClinVar, the base-pair at which it takes place, the distance of the mutation from the PAM, sequence and the clinical condition associated with the variant, if provided by ClinVar. If the distance is 0, the corresponding mutation creates a *de novo* PAM, sequence. Otherwise, the mutation differentiates the seed sequence, whose length is specified by the *variant-window* parameter.

### 2.3 Case studies

**FIGURE 2:**
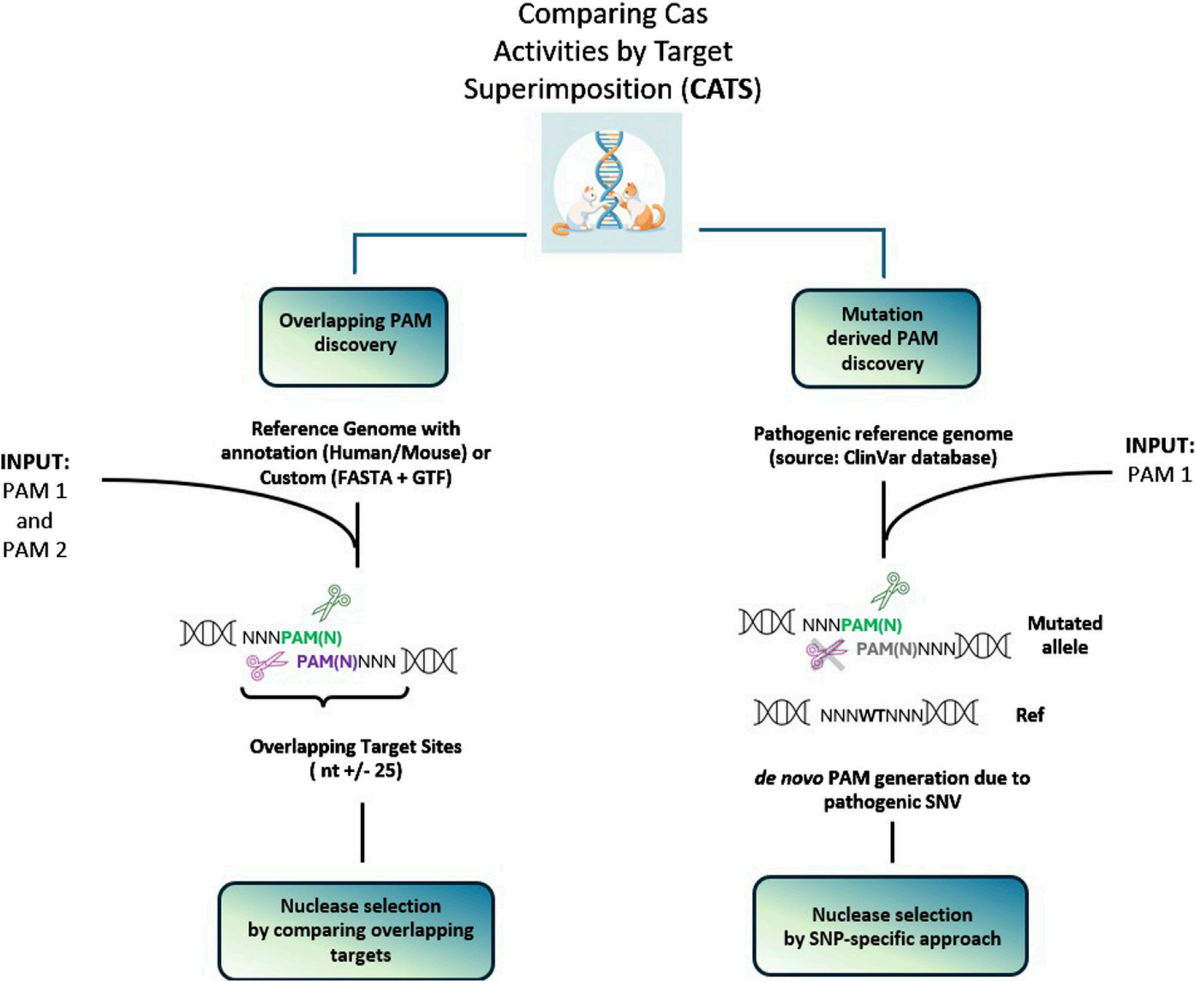
Workflow representations of the two main applications of CATS. The left panel depicts the scenario for finding genomic sites in which two PAM sequences are in proximity or overlapped. The search can be performed only on a selection of genes of interest. The right panel displays the second scenario, in which CATS allows to design an allele-specific approach, enabled by a mutation differentiating the flanking regions of the PAM or creating a *de novo* PAM sequence, not present in the WT.

The following section reports two case studies that present possible applications of CATS and its main features (Figure 2 workflow). The first is the automatic detection of genomic regions where two PAM sequences overlap, providing annotation of pathogenic mutations within the up- and down-stream proximal regions to the overlapping PAM sequences, upon selection by the user. The research can be conducted at a desired genomic location by inserting the name of the gene of interest or by providing a custom FASTA file. The second relevant need addressed by this tool is to help design allele-specific targeting approaches. This is enabled by CATS by ticking the *pathogenic* box, which activates the interaction with ClinVar, thus providing information whether i) a *de novo* PAM is formed as a consequence of a pathogenic SNV ii) SNVs or insertions or deletions (indels) occur within a predefined distance from the PAM. This allows the experimenter to design and test gRNA which can potentially target the disease-carrying allele while sparing the healthy one.

#### 2.3.1 Case study 1: comparing the activity of two Cas9 nucleases with a custom reference genome

**FIGURE 3:**
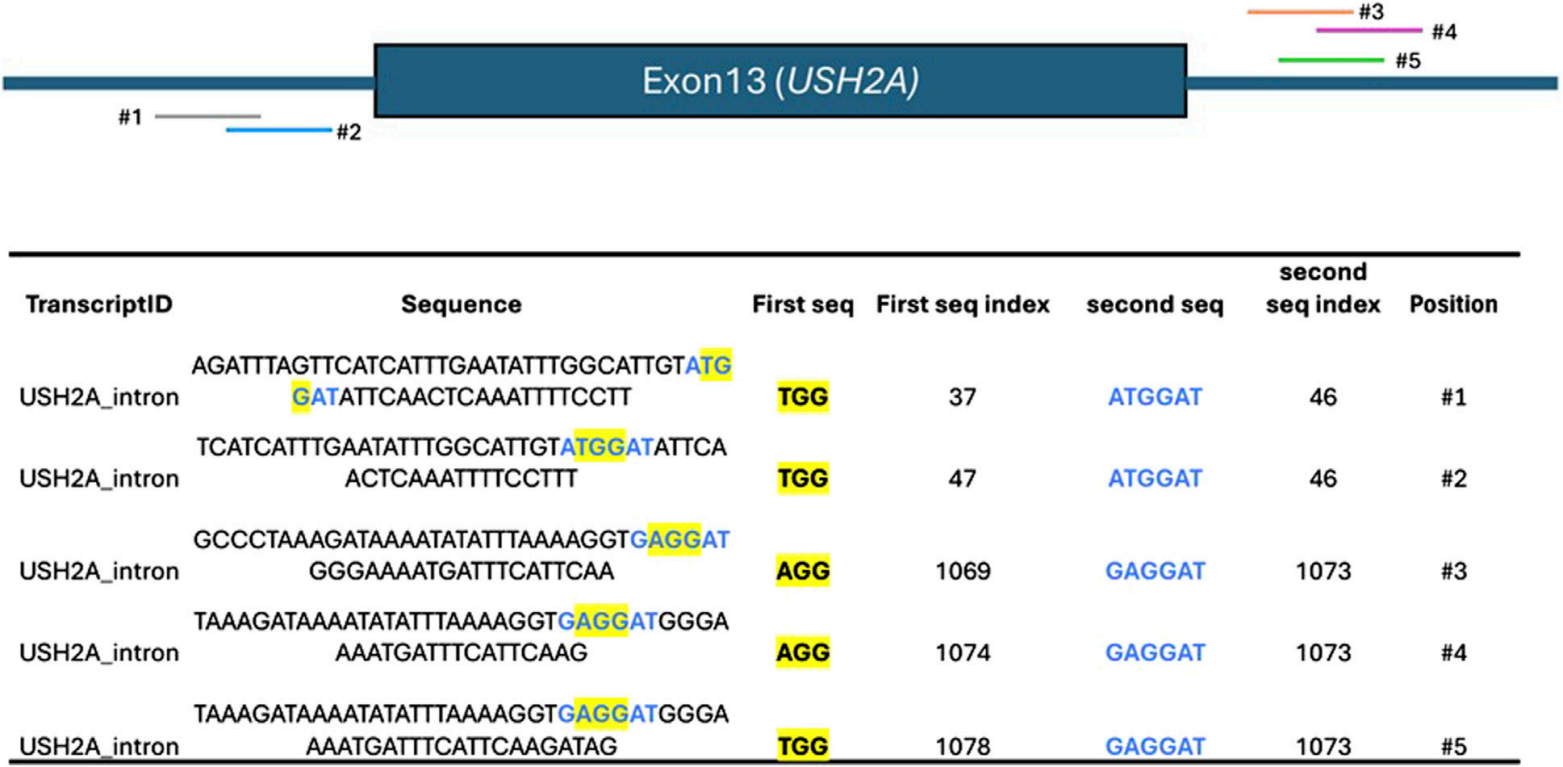
Top: schematics of the Exon13 of *USH2A*, flanked by the intronic sequences, and the positioning of each sequence containing the overlap of the PAMs for SpCas9 and SaCas9. Bottom: output of CATS when instructed to identify the co-occurrences of the PAMs for SpCas9 and SaCas9. The column “*Position*” and the intron’s schematic are added here for the sake of clarity, but they’re not provided by CATS.

When comparing the efficiency of two different nucleases at generating a targeted DNA-Double-Stranded Break (DSB), the genetic landscape of the target site (e.g., GC composition, open vs. closed chromatin) introduces significant bias (Chung et al., 2019; Liu et al., 2016). To account for this, multiple sgRNAs should be tested at different target sites (Degtev et al., 2024; Pedrazzoli et al., 2024). This process is lengthy when done manually, particularly because it often requires the presence of two different PAMs located as close as possible to each other to minimize the bias introduced by the different DNA sequence compositions of the target site. To address this, CATS accepts the PAMs of the two Cas9 nucleases to be compared and the gene of interest (GOI) as input. It then automatically creates a list of targets sharing the two PAMs in close proximity, if not overlapping. To showcase this, we selected *Streptococcus pyrogenes* (SpCas9) (Jinek et al., 2012) - NGG PAM–and *Staphylococcus aureus* (SaCas9) (Friedland et al., 2015) - NNGGRT PAM. These are two of the most widely used Cas9 and oftentimes SaCas9 is being proposed as an alternative to SpCas9 due to its smaller size – 1,053 aa vs. 1,368 – for *in vivo* applications (e.g., AAV-delivery). The target of choice is *Usherin* (*USH2A*) whose mutations are associated with severe forms of *Retinitis pigmentosa* (Pierrache et al., 2016). The most common mutations have been identified in the Exon 13, which leads to a truncated non-functional protein. Currently, the most promising approach is to bypass Exon 13 – exon skipping–to allow for the translation to continue and generate a shorter, yet functional protein. One of the strategies investigated is gene editing, particularly using two sgRNAs to cut within the introns surrounding the mutated Exons to excise it. This approach is being explored both with SpCas9 and SaCas9 (Dai et al., 2023; Schellens et al., 2023). This offers a perfect real-world scenario in which two different Cas9 nucleases are used for the same approach on the same gene. Therefore, we generated a custom FASTA to include the two intronic regions surrounding *USH2A* Exon13 and identified the target sequence where the SpCas9 and SaCas9 PAM, NGG and NGGRT are located in close proximity, thus allowing the experimenter to compare the two Cas9 nucleases in the same genomic context. The results indicated in Figure 3 represent the target sequences which share both PAMs within the introns surrounding Exon13 and that can be potentially used by the researcher to test SpaCas9 and SaCas9 in the same conditions.

#### 2.3.2 Case study 2: allele specific targeting by PAM discrimination

Autosomal dominant diseases are conditions in which the mutation of one of the two alleles is sufficient to cause a pathological effect. These mutations may confer a toxic gain of function that interferes with the function of the healthy copy. In theory, eliminating the allele carrying the mutation may allow the wild-type allele to function properly and ameliorate the condition (Caruso et al., 2022). For the Cas9 to cleave, the PAM must be present immediately next to the intended target. Without this, the Cas9:sgRNA complex cannot undergo the conformational change required to cleave the DNA. Additionally, mismatches present within the first 10-base pairs from the PAM - the so called “seed-sequence” - can dramatically reduce or abrogate altogether the editing efficiency (Liu et al., 2016). Based on this, researchers have investigated whether the mutation may create a *de novo* PAM or a mismatch within the first 3-10 nucleotides from the PAM (seed-sequence) which may be used to specifically target only the mutated allele (Liu et al., 2016; Sabo et al., 2020).

CATS was designed to facilitate the identification of pathogenic SNVs as well as indels that may either result in a *de novo* PAM or disrupt the seed-sequence. This enables the design of gRNAs to specifically target the mutation-bearing allele. For our case study, we first selected *Retinitis Pigmentosa* (RP), a condition for which CRISPR/Cas9 has already been used in studies to knock out a specific allele to improve the phenotype. For this purpose, we selected *NR2E3* (Diakatou et al., 2021). We ran CATS specifying “NGG” as PAM of interest–for SpCas9 as nuclease to use - and set 10 bp in the “Variant Window” parameter, as max distance between the mutation and the PAM, as the “seed-sequence” for SpCas9. In this test, we could identify a number of mutations either generating a PAM *de novo* or occurring within the seed-sequence. Of these, four mutations were associated with Retinal dystrophy. One of them - c.166G>A (G65R) - was used as a proxy to specifically KO the mutated allele to ameliorate RP (Diakatou et al., 2021), confirming that CATS outputs are *bona fide* target for CRISPR/Cas9. The other mutations identified were associated with Enhanced S-cone syndrome, a relatively newly described autosomal recessive retinal generative condition (Iarossi et al., 2022). While not suitable for an allele specific approach, it showcases how CATS can identify potential targets associated with known disease-causing mutations.

Next, we wanted to explore a different target to further validate CATS in implementing allele-specific targeting strategies. For that we selected GATA2 deficiency, an autosomal dominant condition caused by mutations in *GATA2*, a transcription factor essential for the proper function of hematopoietic stem cells, particularly self-renewal and proliferation (Peters et al., 2023). Mutations have been reported throughout the *GATA2* coding sequence, but the Zinc Finger domains 1 and 2 (ZF1 and ZF2) are recognized as mutational hotspots. Currently, the only treatment explored is allogeneic HSC transplantation, which poses significant challenges, such as finding a matching donor. We explored the possibility of designing an allele-specific approach to target the mutated GATA2 allele, with the intent to rescue the phenotype. Following the same procedure as for *NR2E3,* we selected “NGG” as reference PAM and *GATA2* as target gene and we looked for potential mutations allowing us to discriminate between the wild-type allele and the mutated one. As expected, most of the mutations retrieved were located within the two ZF domains. Among them, we identified c.1084C>T (R362X) as suitable target, as it causes a premature stop codon in the ZF2 and has already been reported in multiple patients (Parta et al., 2018). Additionally, we found the missense mutation c.1061C>T which has been previously reported and associated with Acute Myeloid Leukemia (AML) (Hahn et al., 2011).

Overall, these results indicate that the CATS output aligns with findings reported in the literature. Leveraging this information provides a valuable opportunity to identify targets suitable for allele-specific targeting, ultimately contributing to therapeutic strategies for somatic dominant conditions.

## 3 Discussion

In this work, we present Comparing Cas9 Activities by Target Superimposition (CATS), a novel bioinformatic tool that enables comparison of Cas9 nucleases by automated detection of overlapping PAM sequences as well as the identification of targets amenable for allele-specific targeting approaches.

Traditional, manual methods for the curation of PAM sequences and for the identification of overlaps are not only time-consuming but also prone to human error. CATS streamlines this process, providing rapid and accurate results that make the design of CRISPR/Cas9 experiments more efficient and accessible to a broader range of researchers. By automating the detection of overlapping PAM sequences, CATS facilitates the comparison of nucleases characterized by different PAM requirements (e.g., SpCas9 vs. SaCas9: NGG vs. NNGGRT), reducing the bias introduced by the composition of the genetic sequence targeted. Additionally, the tool’s ability to personalize queries and use any reference genome of choice adds a layer of flexibility. While the default settings provide the reference FASTA and annotation files for the human and murine genomes, CATS can work with any other FASTA and GTF files provided by the user, making it a useful tool for researchers working on different models. Moreover, CATS offers the user the possibility to design allele specific targeting approaches, by leveraging the always updated ClinVar database, which is instrumental when the user wishes to devise a strategy where the nuclease targets the pathogenic allele while sparing the healthy one.

We believe that the automation and velocity of CATS is one of its most relevant strengths. This, coupled with its clean and user-friendly interface, ensures that researchers, regardless of their computational expertise, can benefit from the full potential of CATS.

In terms of limitations, one notable constraint is that the default setting works only on protein-coding sequences. This is primarily due to the need for speed in the annotation step, which can be a bottleneck when dealing with large genomic datasets. While the focus on protein-coding regions ensures rapid analysis, it may exclude important non-coding regions that could be relevant for certain studies (e.g., deep intronic cryptic splice sites). However, to mitigate this limitation, we enabled the user to provide a custom FASTA which will allow them to investigate the regions not covered by the default databases included in CATS. It is fair to notice that two previous studies also implemented a feature to allow SNV-targeting via CRISPR, albeit with some differences and in some cases with different goals. The first, CriSNPR (Ansari et al., 2023) is an online-based tool to help design gRNAs for CRISPR-based diagnostics (CRISPRDx). Here the tool searches for SNVs associated with genetic condition as well as presence of infecting agents (e.g., SARS-CoV-2) which can be targeted by a pre-defined set of nucleases. These nucleases are characterized by either a ssDNA or ssRNA collateral cleavage which can be leveraged for developing highly-sensitive detection assays (Zhou et al., 2024). The second, AlPaCas (Rosignoli et al., 2024) similarly to CATS uses the pathogenic SNP as a proxy for allele-specific targeting. By enabling an easier and convenient way to compare Cas9 activities in the same genomic context as well as facilitating the design of allele-specific targeting strategies, CATS can support the implementation of both Cas9 nucleases and Cas9-based approaches for more precise and effective therapeutic solutions for genetic disorders. As the field of genome editing continues to evolve, tools like CATS will play a crucial role in advancing our understanding and application of CRISPR technology, ultimately contributing to the establishment of safer and more efficient genome editing strategies.

## 4 Materials and Methods

### 4.1 Genome parsing

CATS comes with built-in references for both human and murine genomes, including full transcriptome and protein-coding FASTA files retrieved from the latest version of the GENCODE database. However, non-coding variants (such as the ones identified through genome-wide association studies (GWAS)) can be taken into account by providing a custom curated genome source. In this case, the user should provide a FASTA file with the corresponding GTF for the annotations, which should have the same format used by GENCODE, as indicated at the following url: https://www.gencodegenes.org/pages/data_format.html.

As noted above, the sequence search is transcript-agnostic on the WT genome, while transcript-specific information is introduced when the mutated genomes are taken into account. The localization of the sequences along the selected genome source is based on string-matching algorithms, through regular expressions, and the genomic coordinates of the occurrences are derived from the annotation file, in GTF format, along with other features of the transcript. Queries are composed through E-utilities–the dedicated, public API of the NCBI Entrez system–by considering the type of variants, the genes of interest and the pathogenicity. Only pathogenic variants are considered through the analysis. Optionally, the analysis can be restricted to only SNVs.

### 4.2 Strand-handling and PAM orientation

To identify valid CRISPR target sites, the tool requires the protospacer-adjacent motif (PAM) to be specified in the 5′→3′ direction (e.g., 5′-NGG-3′). During the search process, the software scans the input sequence for occurrences of both the PAM and its reverse complement (e.g., 5′-CCN-3′), without reverse complementing the input sequence itself. This approach improves computational efficiency by avoiding the overhead associated with reverse complementing large sequences, while still allowing comprehensive detection of PAM sites on both DNA strands.

When the reverse complement of the PAM (e.g., 5′-CCN-3′) is detected on the plus strand, it indicates that the original PAM (e.g., 5′-NGG-3′) is located on the minus strand, and the corresponding targetable protospacer lies immediately downstream (3′) of the 5′-CCN-3′ motif. Conversely, when the reverse complement motif is found on the minus strand, the corresponding PAM is present on the plus strand, and the targetable protospacer is located immediately upstream (5′) of the motif.

**FIGURE 4:**
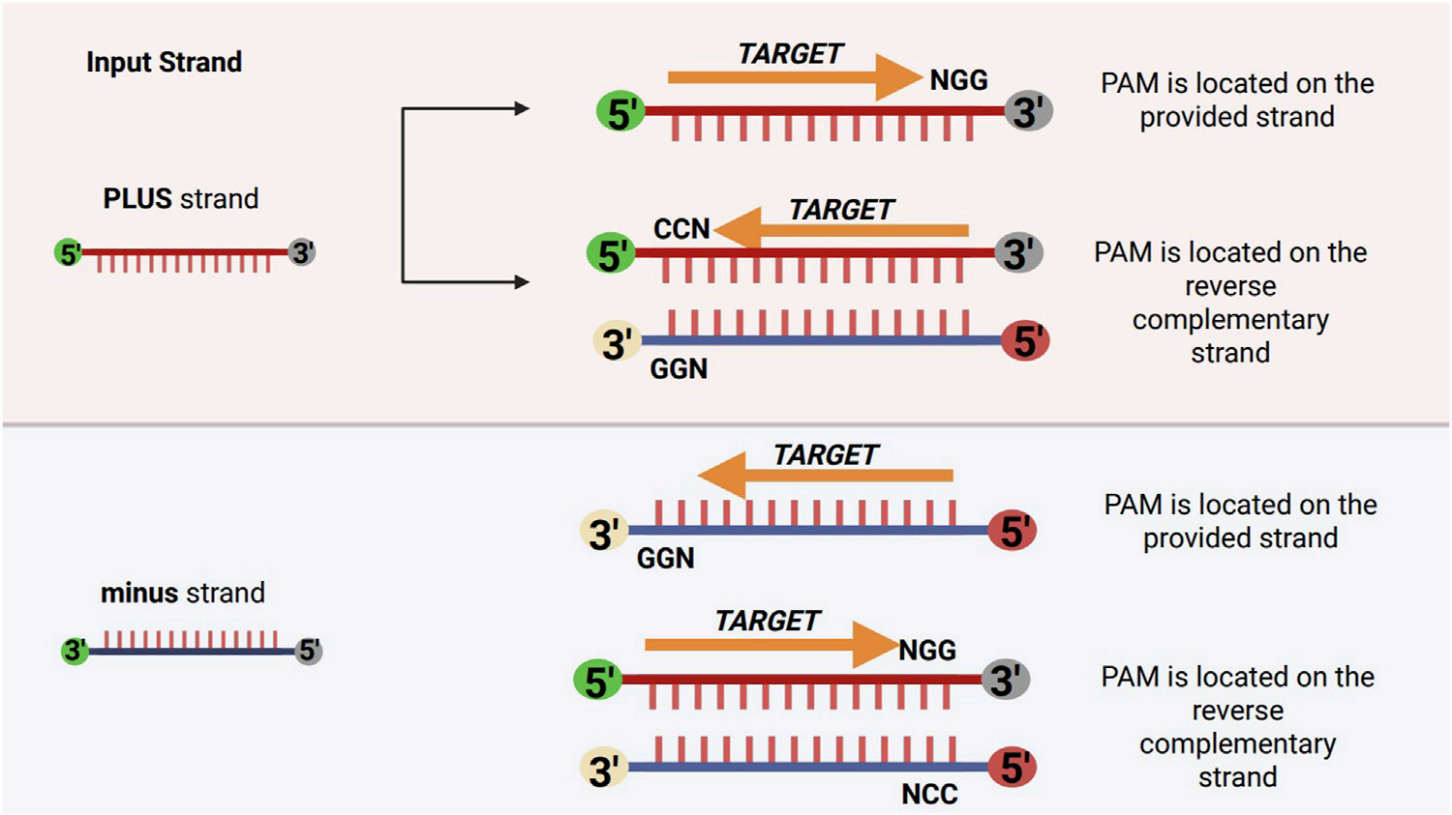
Schematic representation of strand-specific variant window application relative to PAM orientation. For the plus strand, PAMs such as NGG are located on the provided strand, while CCN PAMs are on the reverse complement. For the minus strand, GGN PAMs are on the provided strand, and NCC PAMs are on the reverse complement. The variant window is applied upstream (5′–3′) on the plus strand and downstream (5′–3′) on the minus strand, reflecting the directionality of gRNA-Cas complexes.

In both cases, the software ensures that guide RNAs (gRNAs) are correctly oriented with respect to the PAM and the strand of the target site. This is further clarified and illustrated in Figure 4.

### 4.3 Performance and optimizations

The first time a new annotation file is provided, it will be imported into a local sqlite3 file-based database, an operation that speeds up all its subsequent manipulations. This process could take a while to complete (typically, some tens of minutes), but will take place only the first run.

The major bottleneck of the pipeline is the URL requests to the ClinVar database. Depending on the extent of the considered genome region and the number of known variations in it, the time needed for the annotation process could vary greatly. Another influencing aspect is the speed of the internet connection; therefore, it is advised to run the system on a machine with a wide-band connection available, at least for large queries. Finally, the building of the mutated version of the genome is parallelized, which allows to speed up the system by employing more CPU cores, when available.

We opted for a local installation rather than a web-based service, since it is the most secure choice in terms of cybersecurity. This aspect is particularly relevant considering that many laboratories do not want to disclose the PAMs and, more importantly, the custom genomic data they’re interested in. CATS can be run entirely offline if the user does not require integration with ClinVar. However, when accessing ClinVar data, an active internet connection is necessary. In this case, a stable and fast connection is recommended, as it significantly improves the speed and reliability of information retrieval.

### 4.4 User interfaces and options

CATS is freely and publicly available at https://github.com/Physics4MedicineLab/CATS and is offered through both a user-friendly graphical user interface (GUI) and a command-line interface (CLI). The CLI is accessible for MacOS, Windows and Linux by cloning the GitHub repository indicated above. Linux users may start the GUI from terminal, while we produced standalone executables for MacOS and Windows, implementing directly the GUI. Source code is provided, allowing bioinformaticians to readily integrate the tool into their custom pipelines or even contribute to the project to extend its features.

CATS includes default settings for the human and murine genomes, that can be selected as protein-coding only or with the full-transcriptome. When selected, the tool automatically retrieves the annotation file associated with the reference from GENCODE. The list of genes to be taken into account in a run is provided as the list of their names, divided by a space, or one per line inside a text file. Specific genes of interest can be selected also for custom genomes, if the GTF file with the FASTA annotation is provided as well. For the overlapping-PAM workflow, the window-size parameter defines the maximum distance between two different PAMs for defining a co-occurrence. The number of base-pairs on both sides of a PAM occurrence to report in the results is specified by the num-bases parameter.

When working on human reference, it is possible to search for the occurrences of PAM(s) on the mutated genome by selecting the *pathogenic* box. In this case, the results will list all mutations taking place in the nearing of a PAM or creating a *de novo* PAM. To facilitate finding *de novo* PAM discovery and allele-specific approaches, we included the *variant window* parameter, which defines the maximum distance of the mutation from the PAM for being considered part of the seed sequence (as mentioned above, due to the intrinsic directionality of the gRNA-Cas complexes, the region potentially impacting a given PAM is defined as upstream (5′–3′) on the positive strand and downstream (3′–5′) on the negative strand: variant searches are performed relative to these distinct regions). Even if not mentioned in the case studies, users can provide two PAM sequences even when the *pathogenic* flag is selected. In such a case, the variants reported will be close to the co-occurrences of the indicated PAMs. Finally, a converter is accessible, both from GUI and from CLI, for converting the tabular output into a BED file and viceversa. Table 4 details the options for customizing the analysis by CATS.

**TABLE 4:** List of the options for running CATS. CLI and GUI versions of the tool allow the same level of customization.

CLI command	Description	Type/Extension	Notes
--fasta -f	Reference genome.	FASTA	Mandatory
--gtf -g	The annotation of the reference genome.	GTF	Optional (Mandatory for pathogenicity-associated functionalities)
--gene-list -gl	List of genes to be analyzed, separated by space (or indicated in a txt file, one per line).	String TXT	Advised
--seq1 -s1	Sequence of interest.	String	Mandatory
--seq2 -s2	Second sequence of interest.	String	Optional
--window-size -w	Maximum distance between two PAM sequences for defining their co-occurrency.	Integer number	Optional (it makes sense if two PAMs are indicated) Default: 5
--num-bases	Number of preceding and subsequent bases for each output sequence.	Integer number	Optional Default: 25
--pathogenicity -p	Parse the modified version of the genome, according to the variants retrieved via Clinvar.	Boolean	Optional Default: No
--variant-window -vw	The maximum distance of the mutation from the PAM sequence for being listed in the results.	Integer number	Optional Default: 25
--single-nucleotide-variant -snv	Restricts the analysis only to single-nucleotide variants (SNV).	Boolean	Optional Default: No
--output -o	Output file name.	String	Optional Default: output.csv, output.bed

## Data Availability

CATS is freely and publicly available at https://github.com/Physics4MedicineLab/CATS and is offered through both a user-friendly graphical user interface (GUI) and a command-line interface (CLI). The CLI is accessible for MacOS, Windows and Linux by cloning the GitHub repository indicated above. Linux users may start the GUI from terminal, while we produced standalone executables for MacOS and Windows, implementing directly the GUI.
